# Changes of Field Incurred Chlorpyrifos and Its Toxic Metabolite Residues in Rice during Food Processing from-RAC-to-Consumption

**DOI:** 10.1371/journal.pone.0116467

**Published:** 2015-01-21

**Authors:** Zhiyong Zhang, Wayne W. Jiang, Qiu Jian, Wencheng Song, Zuntao Zheng, Donglan Wang, Xianjin Liu

**Affiliations:** 1 Key Laboratory of Food Quality and Safety of Jiangsu Province / State Key Laboratory Breeding Base / Key Laboratory of Control Technology and Standard for Agro-product Safety and Quality, Ministry of Agriculture of P. R. China, Nanjing, China; 2 Department of Entomology, Michigan State University, East Lansing, MI, United States of America; 3 Institute for the Control of Agrochemicals, Ministry of Agriculture of P. R. China, Beijing, China; Ghent University, BELGIUM

## Abstract

The objectives of this study were to determine the effects of food processing on field incurred residues levels of chlorpyrifos and its metabolite 3,5,6-Trichloro-2-pyridinol (TCP) in rice. The chlorpyrifos and TCP were found to be 1.27 and 0.093 mg kg-1 in straw and 0.41 and 0.073 mg kg-1 in grain, respectively. It is observed that the sunlight for 2 hours does not decrease the chlorpyrifos and TCP residues in grain significantly. Their residues in rice were reduced by up to 50% by hulling. The cooking reduced the chlorpyrifos and TCP in rice to undetectable level (below 0.01 mg kg-1). Processing factors (PFs) of chlorpyrifos and TCP residues in rice during food processing were similar. Various factors have impacts on the fates of chlorpyrifos and TCP residues and the important steps to reduce their residues in rice were hulling and cooking. The results can contribute to assure the consumer of a safe wholesome food supply.

## Introduction

A great concern on toxic pesticides in food has been raised due to pesticides’ negative health and environmental effects. Some food processing treatments result in a significant reduction of pesticide residues in food. Cereal crops comprise more than 60% of agricultural production worldwide. Rice, wheat, and corn are the three most important staple foods. Rice (*Oryza sativa* L.) is the staple food for over half of the world’s population. Its consumption has been increased worldwide over the past decades, as it has become one of the most common foods [1]. In China, rice is the main food and the rice planting acreage accounts for approximately 23% in the world and the production in China is more than 30% of the total world yields, ranking the first in the world [2]. Rice is cultivated in a humid, temperate environment and with a long production period. Therefore, fungi, insects and mites can affect its production. More than 70 insect species have been recorded as rice pests, which are the major constraints on crop yields causing seriously production reduction [3, 4]. For these reasons, several kinds of pesticides are used to protect crops against pest damages. Currently, there are approximately 600 active ingredients and more than 2700 agrochemical products have been registered for controlling rice insects, diseases and weeds in China [5].

Pesticide residues have been found in many raw agricultural commodities (RAC) and processed foods worldwide in the past decades [6–11]. Pesticide residues in rice, brown and polished rice and their risk have been reported [12–13]. The food processing steps may significantly affect the pesticide residues in foods. Scientists and food processors are interested in the effect on persistence of pesticide residues during the commercial food processing procedures and home food processing steps such as washing and cooking [14, 15]. It was observed that washing with water and/or detergent solution decreased the pesticide residues in tomatoes [16]. Cooking (blanching and frying) helped to eliminate most of the pesticide residues from the potato tubers [17]. Successive decreases of pesticide residues occurred during cold storage period [18]. However, microwave cooking could only reduce pesticide residues by a limited amount whereas in-pack sterilization resulted in a complete removal of pesticide [19]. Ultrasonic cleaning for 20 min lowered pesticides by 49.8%–84.4% [20]. Washing with a low concentration of limonene for 5 min was the optimal treatment for elimination of pesticide residues in green pepper, considering the effect and treatment time as well as the cost [21]. Recently, Ling and coworkers observed that the cooking process may result in removal of chlorpyrifos residue in vegetables, more effective than washing [22]. Pazzirota calculated the processing factors of selected pesticides in the vinification process [23]. Different food processing techniques used in commercial or home processing on fruits and vegetables are typically washing, freezing, blanching, peeling, pureeing, maceration, filtration, fermentation, cooking (including roasting, frying, and boiling). In the literatures, most of the studies on the effects on food processing generally attempted to reveal the pesticide residue reductions in one or two processing steps and most work focused on vegetables and fruits with relative high residues. However, it is difficult to obtain a complete processing profile on pesticide change in the crop from harvest to human consumption in terms of processing factors. Because of high production and high consumption of staple foods, it is important to understand the pesticide residue change in rice during the food processing steps. Little work on the pesticide reduction in cereals such as rice during processing steps from harvest to dinning table was reported. It is worthy noting that OECD believes that since most of the studies utilized laboratory spiked samples on food processing, they may not have truly reflected the pesticide distributions in the field crops, for instance, these pesticides may be deposited on the crop surfaces, penetrate inside the crop commodities, or even be chemically bonded to tissues of the field crops [24].

Chlorpyrifos, CAS number 2921-88-2, IUPAC name O,O-diethyl O-3,5,6- trichloropyridin-2-yl phosphorothioate, is an organophosphate insecticide, acaricide and miticide and is used to control foliage and soil-borne insect pests on rice crops. Chlorpyrifos can cause human health concerns such as nausea, dizziness, confusion, and at high exposures, it may cause lung cancer, respiratory paralysis and death [25, 26]. The metabolite, 3,5,6-Trichloro-2-pyridinol(TCP), CAS number 6515-38-4, is a toxic chemical which was found to lower testosterone levels in men [27].

The aims of this study were (1) to assess field incurred chlorpyrifos and its toxic metabolite 3,5,6-trichloro-2-pyridinol (TCP) residues in grain and straw under the conditions of critical good agriculture practice (cGAP), (2) to investigate the continuous changes of their residues in rice during rice processing steps from harvest to dinning table, and (3) to determine the processing factors (PFs) which can be used to estimate the exposure level of pesticide residues during the complete process from the harvested raw rice crop (raw agricultural commodity, RAC) to cooked rice at dining table (for human consumption), so-called from-RAC-to-consumption (or from-RAC-to-dining-table).

## Materials and Methods

### Materials

HPLC-MS/MS analysis was performed on a 1200SL HPLC system (Agilent Technologies, Santa Clara, CA, USA) coupled to an Agilent G6410A triple quadrupole mass spectrometer. The system was controlled by MassHunter software. The analytical column was an Agilent ZORBAX SB-C18 (2.1 × 150 mm, 5 μm) analytical column with a guard column (Agilent ZORBAX SB-Aq Narrow-Bore Guard Column, 2.1 × 12.5 mm, 5 μm). Other equipment included an IKA high-speed homogenizer (T-18; IKA Werke, Staufen, Germany), a shaker (PYB, China Academy of Science Wuhan Science Equipment Factory, China), a centrifuge (Beckman Coulter, Avanti J-30I, USA), a internal pump backpack sprayer (JACTO-HD 400, Maquinas Agricolas Jacto S.A., Brazil), a rice cleaner (MB-RC71, Yamamoto Co., Ltd., Japan) and a husker (Satake THU35B, Satake Manufacturing (Suzhou) Co. Ltd, China).

Pesticide standards, chlorpyrifos (99.5%, w/w) and TCP were purchased from Sigma-Aldrich (Shanghai, China). Chlorpyrifos 20% emulsifiable concentrate (Dursban 20% EC) was purchased from Shanghai HuiKwang Chemical Co., Ltd. (Shanghai, China). Acetonitrile was purchased from Merck (Darmstadt, German). Purified water was prepared by a Milli-Q water purification system (Millipore, Bedford, MA, USA). All other solvents and reagents used in the present study were of HPLC grade or analytical grade, unless otherwise noted.

A stock standard solution of chlorpyrifos or TCP was prepared by weighing chlorpyrifos or TCP and dissolving it in acetonitrile. An initial work standard solution was prepared by mixing same portions of the chlorpyrifos and TCP stock solutions. The other stock standard solutions (0.01, 0.05, 0.1, 0.5, and 5 μg mL^−1^) were prepared by diluting the initial working solution or another working solution with acetonitrile. All standard solutions were maintained in amber bottles at 4°C.

### Field Trials and Sampling

A field study was conducted in paddies in Nanjing City, Jiangsu Province (32°-04′ north latitudes and 118°-78′ east longitudes) in 2012. No specific permissions were required for this location. All field studies did not involve endangered or protected species. This study was carried out according to “Standard Operating Procedures on Pesticide Registration Residue Field Trials” issued by the Institute of the Control of Agrochemicals, Ministry of Agriculture, P. R. China [28]. Details about applications referenced to the chlorpyrifos label were summarized as follows: rate of application 720 g a.i. ha^−1^ (gram active ingredient per hectare), two applications, and pre-harvest interval (PHI) 28 days [29]. The applications were carried out using a JACTO-HD 400 sprayer. One control and three replicates of treated samples with an area of 60 m^2^ of each sample. Rice plants with grains were sampled. In the control plot, no pesticide was sprayed during the experiment.

### Design of Rice Processing Procedure

**Figure 1:**
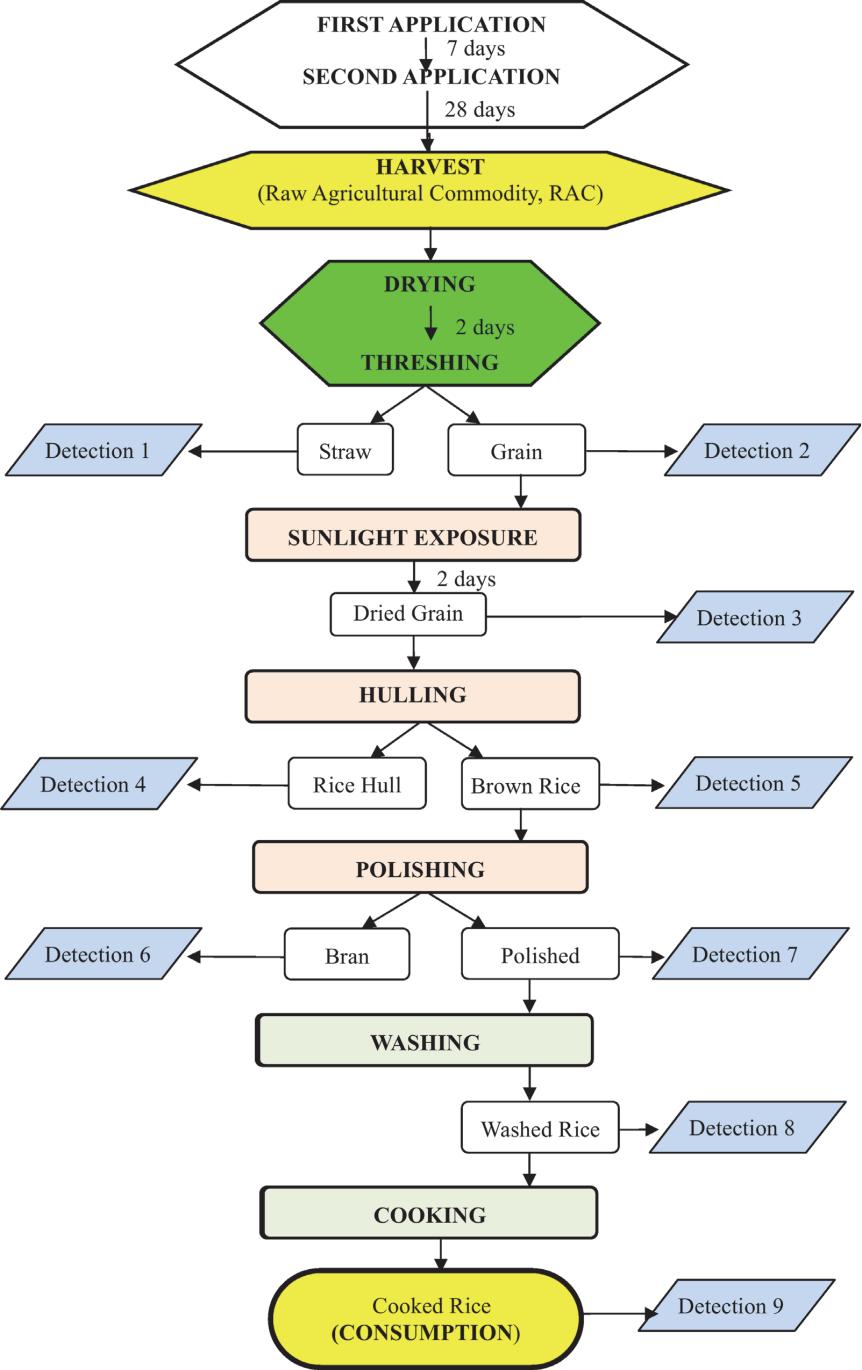
Scheme of processing rice from-harvest-to-dining-table.

The experiment was designed to monitor the residues during the rice processing steps that were actually used in field drying under the sun, industrial hulling/polishing processing steps and home cooking. For example, after harvesting, drying, and threshing, grains were subjected to the primary milling operation which includes de-husking (hulling) as well as the removal of bran layers (polishing) before it is consumed [1]. Therefore, the residues of chlorpyrifos and TCP in rice at different processing steps were determined to evaluate the effects of sunlight exposure, hulling, polishing, washing, and cooking on chlorpyrifos and its metabolite TCP in rice from-harvest-to-dining-table. The steps of experiment were described in Fig. 1.

The rice samples (aboveground part including straws and grains) were harvested at PHI 28 days. The harvested rice plants (grains and straws) were dried under sunlight for two days on the cement floor. The samples were turned over every 3 hours. The average temperature, relative humidity, and wind speed were 32°C, 50%, and 3 m s^−1^, respectively. The samples after sunlight were separated into straws and grains using a threshing machine. Next, grains were sunlight exposed on a plastic pan for two days, and this drying step is necessary for rice storage, especially for an extended period of time. Then, grains were hulled using a hulling machine and rice husk and brown rice were obtained. The brown rice was polished using a polishing machine. The polished rice was washed 3 times using tap water (rice: water = 1:1, w/w) and each washing was 10 seconds with constant stirring. Finally, the washed rice mixed with water (rice: water = 1:1, w/w) was cooked using a rice cooker. The cooked rice is generally for human consumption. The residues of chlorpyrifos and TCP in straw, grain, rice husk, brown rice, bran, polished rice, washed rice, and cooked rice were analyzed at the processing steps.

### Analytical Methods

In this study, the analytical method for determining the chlorpyrifos and TCP residues in rice samples was a QuEChERS method modified from reference methods [30–32].

#### Extraction

Rice sample (5.0 g) was weighted into a polypropylene bottle. An extraction solvent (10 mL of acetonitrile) was added and the sample was macerated using the homogenizer with 1 min. The probe of the homogenizer was rinsed with a portion of 5.0 mL of the extraction solvent. The rinsate and the extraction solvent were combined in the centrifuge tube. The sample was centrifuged at 5000 rpm for 5 min. The supernatant was used for clean-up.

#### Clean-up

The supernatant (2 mL) was transferred into a 15 mL centrifuge tube containing PSA (100 mg), ODS-C18 (100 mg) and florisil (100 mg). The sample mixture was vortexed for 2 min and centrifuged at 5000 rpm for 2 min. After centrifugation, the supernatant was filtered using a 0.22 μm nylon filter into an autosampler vial.

#### LC-MS/MS Analysis

According to the SANCO/10684/2009 guideline calibration curves were constructed by plotting 0.01–5.0 μg mL^−1^ of chlorpyrifos and TCP standards injected versus peak area. From the calibration curves the concentrations of chlorpyrifos and TCP in samples were determined.

LC-MS/MS was conducted with the Agilent LC/MS/MS. The parameters were as follows. A gradient elution program was used in this analysis: mobile phase A: water containing 0.1% of formic acid (v/v); mobile phase B: acetonitrile containing 0.1% of formic acid (v/v); injection volume 10 μL; flow rate 0.4 mL min^−1^; mobile phase gradients of binary pump: 10% B (0–1.50 min), 95% B (1.51–4.50 min), 95% B (hold, 4.51–6.00 min), and 10% B (6.01–7.00 min); and the parameters were as follows: gas flow 10 L min^−1^; gas temperature 350°C; nebulizer pressure 45 psi; and capillary voltage 4000 V. In order to achieve the highest sensitivity, the fragment, voltage and the collision energy were optimized. For chlorpyrifos and its metabolite TCP, the other MS instrument was operated as conditions summarized in Table 1.

**Table 1:** Retention time, monitoring ions and collision energy of chlorpyrifos and TCP in the multiple reaction monitoring mode.

Compound	Electrospray ionization	Retention Time (min)	Precursor ion (*m/z*)	Product ion (*m/z*)	Collision energy (V)
Chlorpyrifos	positive	3.9	350.1	97, 198^1^	30, 20^1^
TCP	negative	3.0	195	35, 37^1^	15, 15^1^

^1^ Quantitative ion.

### Processing Factor

Processing factor (*PF*) for a rice processing step is calculated by

$$PF=C_{\text{post}}/C_{\text{pre}}$$ (Eq. 1)

where *C*_post_ and *C*_pre_ are the pesticide residue concentrations (mg kg^−1^) for post-processing to pre-processing, respectively.

For the multiple steps, the cumulative processing factor (*PF*_cum_) is calculated by

$$PF_{\text{cum}}=PF_{1}\cdot PF_{2}\cdot \ldots \cdot PF_{\text{n}}=C_{\text{n}}/C_{0}$$ (Eq. 2)

where *PF*_n_ is the individual processing factor for step n, and *C*_n_ and *C*_0_ are the pesticide concentrations after the n-th processing step and before the initial processing step, respectively. If a *PF* is lower than 1, it means a reduction in the pesticide, indicating the processing step is effective to decreasing the risk in the regulatory practice, regardless of changes in volume or weight for the commodity [33]. However, if the *PF* is greater than 1, possibly due to concentration (such as drying), such a higher concentration of the pesticide raises the risk concerns.

### Statistical Analysis

Sample analyses were performed on triplicate samples and statistical data are presented as mean ± standard deviation (SD), which were subjected to analysis of variance (ANOVA). The data were tested for homogeneity of variances at a significance level of P < 0.05 and probability values of less than 0.05 were considered as statistically significant (one-way ANOVA). Significant means were subjected to analysis by Duncan’s multiple range test (*p* < 0.05). For pair-wise comparisons between unprocessed samples and different processed samples paired t-test was performed. SPSS version 11.5 (SPSS Inc., Chicago, IL, USA) was used for statistical analysis.

## Results and Discussion

### Linearity, Recoveries, and LOQ

The calibration curves were obtained by plotting the peak area against the concentrations of chlorpyrifos/TCP of the corresponding calibration standard solutions. Linearity regressions of *y* = 120.96 *x* + 56.15 (R^2^ = 0.9998) and *y* = 110.43 *x* − 24.31 (R^2^ = 0.9994) for chlorpyrifos and TCP, respectively, were used. The methodology for the determination of chlorpyrifos and TCP in the samples demonstrated that chlorpyrifos and TCP can be accurately determined at a limit of quantitation (LOQ) of 0.01 mg kg^−1^. The LOQ is defined as the lowest fortification level at which acceptable recovery data are obtained [28]. The limit of detection (LOD) of the method is defined as the lowest calibration standard chromatographed having a peak area equivalent to, or better than three times the baseline noise [27]. In this study the LOD was found to be 0.1 ng. The spiked levels were set to be 0.01, 0.1 and 2.0 mg kg^−1^ for the samples. The mean recovery percentages of chlorpyrifos ranged from 80.7% to 102.8% with relative standard deviations (RSD) from 1.1% to 9.6%, respectively. TCP’s recoveries ranged from 82.1% to 103.3% with RSDs from 1.4% to 9.7%, respectively. As can be seen, the method was suitable for the determination of chlorpyrifos and TCP residues in all rice samples [27].

### Field Incurred Chlorpyrifos and TCP residues in Rice

Aboveground plants (including straw and grain) were harvested at 28 days after the last application (PHI 28 days). Then the harvested rice plants were separated to straw and grain by a threshing machine after sunlight exposure for 2 days. The chlorpyrifos and TCP residues in straw and rough rice were detected. Chlorpyrifos residues in straw and rough rice were found to be 1.27±0.13 and 0.41±0.08 mg kg^−1^, respectively (Table 2). TCP residues in straw and rough rice were found to be 0.093±0.012 mg kg^−1^ and 0.073±0.012 mg kg^−1^, respectively. Data showed that the residues in straw were higher than in grain. These results were consistent with the previous findings for the chlorpyrifos residues in paddy system [4].

**Table 2:** Chlorpyrifos and TCP residues in rice during food processing.

Processing Step	Sample	Detection Number^1^	Chlorpyrifos (mg/kg)^2^	TCP (mg/kg)^2^
Harvest	Straw (initial)	1	1.27 ± 0.13	0.093 ± 0.12
	Grain (initial)	2	0.41 ± 0.08	0.073 ± 0.012
Drying	Dried grain	3	0.38 ± 0.03	0.071 ± 0.011
Hulling	Hull	4	1.18 ± 0.05	0.26 ± 0.11
	Brown rice	5	0.19 ± 0.04	0.035 ± 0.013
Polishing	Bran	6	1.11 ± 0.12	0.70 ± 0.08
	Polished rice	7	0.11 ± 0.04	0.018 ± 0.004
Washing	Washed rice	8	0.05 ± 0.02	0.01 ± 0.002
Cooking	Cooked rice	9	ND (0.007)^3^	ND (0.002)^3^

^1^ Detection number corresponding to the Detection number shown in Fig. 1

^2^ Standard deviation (±SD) calculated from three replicates of samples (*n* = 3)

^3^ ND: non-detectable (i.e., < 0.01 mg/kg); The values in brackets estimated to be approximately 0.007 and 0.002 mg/kg for chlorpyrifos and TCP, respectively, based on peak areas obtained.

Table 3 lists the Maximum Residue Limits (MRLs) established in various countries and Codex Alimentarius Committee [34–39]. As can be seen, the chlorpyrifos residue from this study was 0.41 mg kg^−1^, which is below the MRLs set in China and by CAC, but higher than MRLs set by EU, Australia and Korea.

**Table 3:** Maximum residue limits (MRLs) for chlorpyrifos in rice (mg/kg).

Pesticide	Nation, Region or Organization					
	China^1^	Europe^2^	CAC^3^	Australia^4^	Korea^5^	Japan^6^
Chlorpyrifos	0.5	0.05	0.5	0.1	0.1	0.1(brown rice)

^1^ GB 2763–2012. China’s National Food Safety Standard-Maximum Residue Limits for Pesticides in Food.

^2^ European pesticide residues and maximum residue levels database, Regulation (EC) No 396/2005.

^3^ Codex pesticide residues limits in food and feed database.

^4^Australian agricultural and veterinary chemicals code instrument No. 4 (MRL Standard).

^5^ Korea MRLs for pesticide.

^6^The Japanese positive list system for agricultural chemical residues in foods.

#### Sunlight exposure

The grain obtained by the threshing was sunlight exposed for 2 days before the residues were analyzed. Chlorpyrifos and TCP residues grain were changed from 0.41±0.08 mg kg^−1^ to 0.38±0.03 mg kg^−1^ and from 0.073±0.012 mg kg^−1^ to 0.071±0.011 mg kg^−1^, respectively (Table 2). The results showed that sunlight exposure could reduce a small amount of chlorpyrifos and TCP in grain. The amount of chlorpyrifos residues in rice (via simulative spiked, but the level was unknown) were reduced from 6% to 20% after sunlight exposure for 1 to 3 days [40]. Degradation trends of chlorpyrifos in rice (brown) in this study were consistent with those found in the literature. However, for grape, the raisins obtained by sun-drying did not present quinoxyfen residues, whereas those obtained by oven-drying showed the same amount of residues as in the fresh grapes [41]. This is probably because the drying step involves loss of water content resulting in higher concentrations of pesticides while the degradation of the pesticides proceeds.

#### Hulling

Hulling is essential to rice processing. The rough rice (grain) obtained by sunlight exposure was hulled by the hulling machine. The weight ratio of rice husk to brown rice was approximately 2:8 (w/w) in this study. Then chlorpyrifos and TCP residues in rice husk and brown rice were detected. After hulling, chlorpyrifos residues in rice husk and brown rice were found to be 1.18±0.05 mg kg^−1^ and 0.19±0.04 mg kg^−1^, and TCP residues were found to be 0.26±0.11 mg kg^−1^ and 0.035±0.013 mg kg^−1^, respectively (Table 2). These data indicated that the chlorpyrifos and TCP residues in rice husk were significantly higher than those in brown rice, i.e., chlorpyrifos is approximately 10 times higher in hull based on the weight ratio (absolute mass). In this study, the chlorpyrifos residue in brown rice is 0.26 mg kg^−1^, exceeding the Japanese MRL, 0.1 mg kg^−1^ (Table 3). The Maximum Residue Limits (MRLs) in either brown rice or rice husk have not been set by other nations or CAC.

#### Polishing

Polished or white rice is the most common form of rice and it’s consumed in many countries or regions. Milling can remove the outer bran layer of the brown rice and it’s the primary difference between the brown rice and polished rice. The brown rice (chlorpyrifos and TCP residues were 0.19±0.04 mg kg^−1^ and 0.035±0.013 mg kg^−1^, respectively) was separated to bran and polished rice by a milling machine. The weight ratio of bran to polished rice was approximately 0.08:1 (w/w) in this study. Chlorpyrifos residues in bran and polished rice were 1.11±0.12 mg kg^−1^ and 0.11±0.01 mg kg^−1^, and TCP were 0.70±0.08 mg kg^−1^ and 0.018±0.004 mg kg^−1^, respectively (Table 2). The data demonstrated that chlorpyrifos and TCP residues in bran were much higher than those in polished rice.

#### Washing

Washing is the most common and straightforward step of household rice processing. Before cooking, polished rice is usually washed by tap water two or three times. In this study, the polished rice (chlorpyrifos and TCP residues were 0.11±0.04 mg kg^−1^ and 0.018±0.004 mg kg^−1^, respectively) was washed 3 times with same amount of tap water (1:1, w/w). Chlorpyrifos and TCP residues in washed polished rice were analyzed after air drying. The data showed that chlorpyrifos and TCP residues in washed polished rice were 0.05±0.02 mg kg^−1^ and 0.01±0.002 mg kg^−1^, respectively (Table 2). In the literature, it was reported that washing with tap water, different detergents, salt solutions (NaCl), and vinegar (acetic acid) may decrease the chlorpyrifos pesticide in cabbage by between 10% and 70% [42]. Recently, another report demonstrated that washing with different concentrations of limonene caused 20%-90% loss of the chlorpyrifos residues in green peppers [21]. However, Burchat et al. observed that washing did not significantly decrease the amounts of organophosphorus insecticides in carrots [43]. It is understood that the loss of pesticide primarily depends upon the physicochemical property, such as aqueous solubility and acidity, etc.

#### Cooking

Cooking is an important and the final food processing step. The polished rice where chlorpyrifos and TCP residues were 0.05±0.02 mg kg^−1^ and 0.01±0.002 mg kg^−1^, respectively, was cooked by an automatic rice cooker. The weight ratio of polished rice to distilled water was 1:1. The residues of chlorpyrifos and TCP residues in cooked rice were not detected. Their residues in cooked rice below 0.01 mg kg^−1^, but the calculations indicate that the residues of chlorpyrifos and TCP residues could be approximately 0.007 and 0.002 mg kg^−1^, respectively, based upon the peak areas (Table 2). Cooking and storage processing could reduce the residues of five pesticides of dichlorvos, chlorpyrifos-methyl, malathion, fenitrothion, and bromide in rice in post-harvest applied [44].

### Processing factors

**Figure 2:**
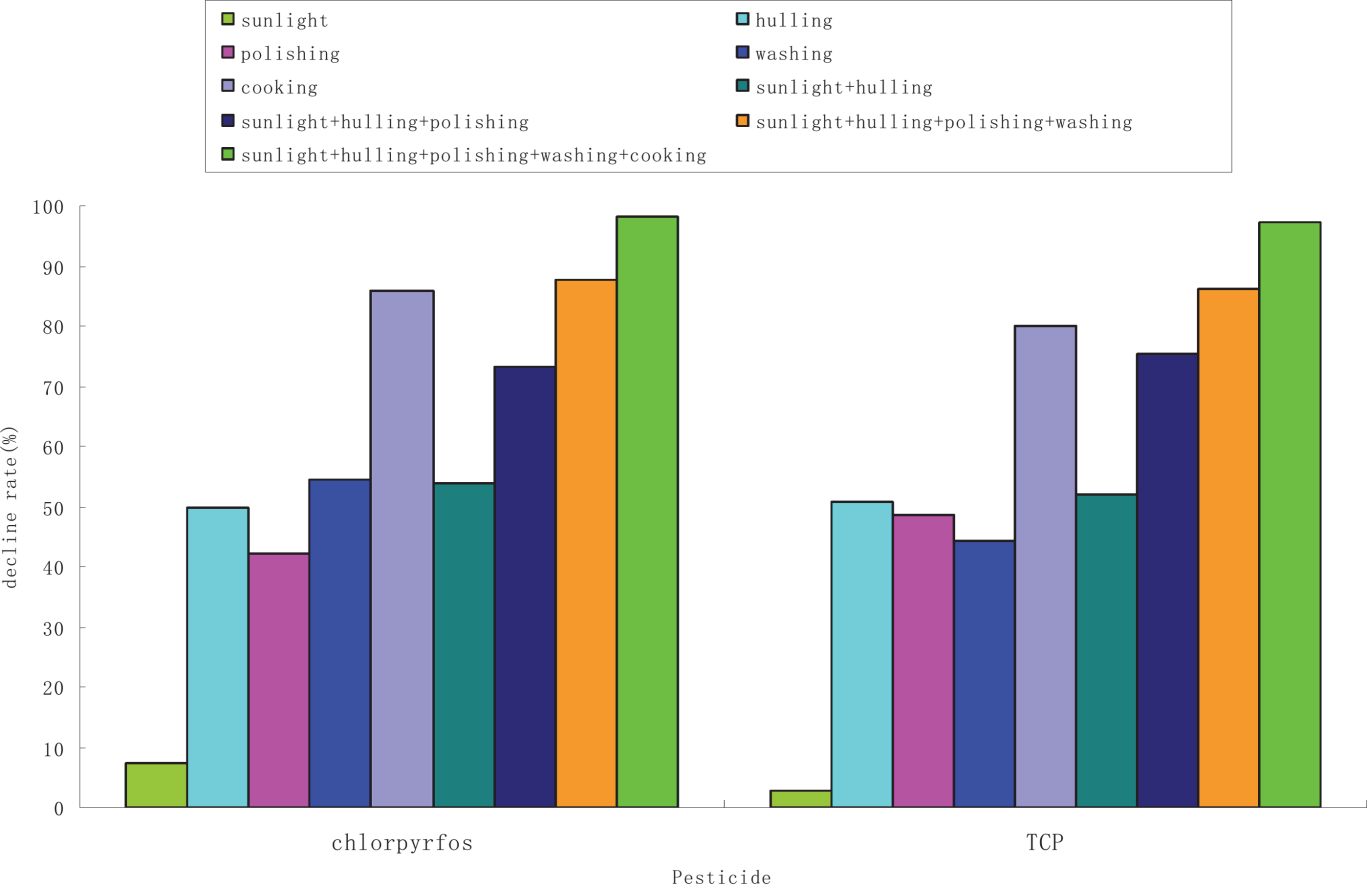
Declines of chlorpyrifos and its toxic metabolite TCP residues in rice during food processing.

Processing factors are given in Table 4. For individual steps, the *PF*s of chlorpyrifos and TCP ranged from 0.45 to 0.93 and 0.49 to 0.97, respectively. For combination steps, the *PF*s of chlorpyrifos and TCP ranged from 0.12 to 0.93 and 0.14 to 0.97, respectively. The data show that at drying step (2-day sunlight exposure), *PF* was 0.93 and 0.98 for chlorpyrifos and TCP, respectively, indicating the drying does not affect the residues significantly (*p* < 0.05). The data show that the *PF*’s of the next three processing steps (hulling, polishing, and washing) are close to 0.5 for both chlorpyrifos and TCP, significantly lower than those for drying by sunlight (*p* < 0.05). The data suggest that, in this study, each of the hulling, polishing, and washing processing steps decreases the residues by approximately 50%, were significantly reduced by hulling, polishing, washing and cooking had significant effects on their residues in rice (*p* < 0.05). As shown in Table 2 and Fig. 2, the chlorpyrifos and TCP residues in cooked rice were not detectable (below 0.01 mg kg^−1^, but can be estimated to be approximately 0.007 and 0.002 mg kg^−1^, respectively) based on the peak areas. Therefore, *PF* and *PF*_cum_ are estimated to be approximately 0.11 and 0.01 for chlorpyrifos and 0.22 and 0.002 for chlorpyrifos, respectively, after the rice was cooked. This indicates that the cooking step is the critical processing step to control the chlorpyrifos and its toxic metabolite TCP residues in rice during food processing. The cumulative *PF*s are below 0.01, indicating that the residues in rice by human consumption were significantly lower than the levels before food processing (Table 3).

**Table 4:** Processing factors (*PF*_s_) for individuals and cumulative (*PF*_cum_).

Processing Step	Sample matrix	Chlorpyrifos^1^		TCP^1^	
		*PF* ^2^	*PF*_cum_ ^2^	*PF* ^2^	*PF*_cum_ ^2^
Harvest	Grain (initial)	-	-	-	-
Drying	Dried grain	0.93±0.11 a	0.93±0.07 a	0.97±0.08 a	0.97±0.11 a
Hulling	Brown rice	0.50±0.01 b	0.46±0.04 b	0.49±0.01 b	0.48±0.03 b
Polishing	Polished rice	0.58±0.03 b	0.27±0.03 c	0.51±0.02 b	0.25±0.06 c
Washing	Washed rice	0.45±0.03 b	0.12±0.01 d	0.56±0.04 b	0.14±0.08 d
Cooking	Cooked rice	0.11^3^ c	0.01^3^ e	0.22^3^ c	0.002^3^ e

^1^ The letters (a, b, c, d, and e), which are followed by the *PF* and *PF*_cum_ values, are significantly (*p* < 0.05) different (Duncan’s multiple range test);

^2^ *PF* and *PF*_cum_ are the individual and cumulative processing factors, respectively.

^3^ At the cooking step, the residues < 0.01 mg/kg (but appeared to be approximately 0.007 and 0.002 mg/kg, based upon the peak areas) and thus, *FP*s and *PF*_cum_ estimated in the same manner of other processing steps.

## Conclusions

One of the most common routes of pesticide exposure to consumers is via food consumption. It is important for the consumer to understand the intake of pesticide residues. The pesticide residues in food commodities are typically reduced during food processing. In this study, various food processing factors such as sunlight exposure, hulling, polishing, washing, and cooking on rice were determined for field incurred chlorpyrifos and its toxic metabolite TCP. For example, this work describes the profile of the chlorpyrifos and TCP residues from harvest to the dinner table (from-RAC-to-consumption). Data showed that chlorpyrifos residues in harvested rice were found to be 0.41±0.08 mg kg^−1^ under the cGAP conditions. It was below the MRLs set by China and the CAC, but exceeded the EU’s and Korean regulated limits. Their residues were declined during these food processing and were below 0.01 mg kg^−1^ in cooked rice after continuous food processing. Each factor had an impact on the fates of chlorpyrifos and TCP residues after each individual processing step. The data reveal that the important step to reduce pesticide residues in rice was cooking. It can be concluded that food processing such as cooking is necessary to reduce the risk of pesticide as the intake of pesticide residues in rice is significantly decreased.

Exposure assessment of pesticide residues is necessary in order to reach a conclusion on the acceptability of proposed MRLs and the underlying GAP from a public health point of view. The dietary intake of a pesticide residue in a given food is obtained by multiplying the residue level in the food by the amount of that food consumed. The total dietary intake of the pesticide residue is then obtained by summing the intakes of all foods containing the residue. As can be seen in this study, a series of food processing resulted in the decrease of chlorpyrifos and TCP residues. As a result, food processing factors (PFs) should be considered when conducting an assessment of pesticide residues in food and establishing MRLs for processed commodities.
